# The Impact of LED Light Spectra on the Growth, Yield, Physiology, and Sweetness Compound of *Stevia rebaudiana*

**DOI:** 10.3390/biology14020108

**Published:** 2025-01-21

**Authors:** Naofel Aljafer, Abdullah Alrajhi, Toby Anderson von Trampe, William Vevers, Sophie Fauset, Hail Zuhir Rihan

**Affiliations:** 1 School of Biological and Marine Sciences, University of Plymouth, Portland Square Building, Drake Circus, Plymouth PL4 8AA, UK; 2 The National Research and Development Center for Sustainable Agriculture (Estidamah), Riyadh Techno Valley, King Saud University, Riyadh 12373, Saudi Arabia

**Keywords:** *Stevia rebaudiana*, light-emitting diode, controlled-environment agriculture, UV, blue light wavelength (435 nm), stevioside, reb A, HPLC

## Abstract

This study investigated the effects of several light spectra on *Stevia rebaudiana,* analysing growth parameters, yield, and physiological responses within a controlled-environment agriculture (CEA) system. The experimental design involved different light treatments, including specific combinations of blue (435 nm and 450 nm), red (663 nm), and ultraviolet (UV) wavelengths (365 nm), to determine their impact on morphological development and biochemical properties, particularly focusing on the production of the sweetening compounds stevioside and rebaudioside A. *Stevia rebaudiana* plants cultivated from cuttings sourced from a reputable UK nursery (Gardener’s Dream Ltd., Glasgow, UK) were subjected to these spectral treatments over a period of five weeks under vertical farming conditions. Physiological measurements, such as chlorophyll fluorescence (*Fv*/*Fm*), stomatal conductance, and leaf temperature, were recorded, alongside growth metrics (plant height, leaf area, and biomass). This study also incorporated high-performance liquid chromatography (HPLC) to quantitatively analyse the influence of the light treatments on the sweetener concentration. The results demonstrated that targeted LED spectra, particularly those that include UV light and blue light (435 nm), significantly nhanced both the quantitative and qualitative attributes of stevia growth, indicating that strategic light management can markedly improve the nutritional and commercial yields of *Stevia rebaudiana*. This research contributes to the optimisation of light conditions in vertical farming systems, aiming to enhance agricultural efficiency and reduce the reliance on imported stevia by maximising local production capabilities.

## Simple Summary

*Stevia rebaudiana*, a plant widely known for its natural sweetening compounds, is gaining global attention due to the increasing demand for low-calorie sweeteners. This study examines how specific LED light spectra affect stevia’s growth, physiology, and the concentrations of its two key sweetening compounds, stevioside and rebaudioside A. By testing various combinations of blue, red, ultraviolet (UV), and white light, the optimal light conditions are identified that enhance growth and sweetness production in a controlled environment. The findings highlight how advance LED lighting can improve local stevia production, reduce the dependence on imports, and contribute to more sustainable agriculture.

## 1. Introduction

*Steviarebaudiana* Bertoni (stevia), a perennial plant belonging to the Asteraceae family, is native to the highlands of Brazil and Paraguay [1]. The steviol glycoside (SG) compounds found in flowers, stems, and leaves form the basis for zero-calorie sweeteners and have gained widespread global popularity due to the absence of harmful side effects commonly associated with synthetic artificial sweeteners [2,3]. In 2014, the demand for stevia extracts was 5100 tonnes, valued at USD 338 million, and it is estimated that it will reach 8100 tonnes with a global market value of USD 554 million by the end of 2024 [4,5,6,7]. In developed and developing Asian countries, such as Malaysia, dried stevia leaves and extracts have gained traction as a natural low-calorie sweetener and herbal supplement, mainly due to growing concerns about obesity and diabetes [8,9]. Currently, many countries rely on imports of *S. rebaudiana* in the form of unprocessed leaves and processed products from China, India, and South America, mainly owing to the non-ideal photoperiod conditions for local cultivation [10]. To reduce the dependency on imported products and meet the increasing local demand, there is a need to enhance the productivity of *S. rebaudiana* beyond its typical altitude range. This can be achieved by optimising the cultivation environments in controlled-environment agriculture (CEA) systems [8,10].

Vertical farming represents a revolutionary agricultural model, reshaping cultivation methodologies. Through the vertical stacking of crops in multi-tiered structures, this approach optimises spatial utilisation, yielding higher crop outputs per unit area compared to conventional horizontal farming. Notably, its environmental benefits include reduced land, water, and pesticide usage, alongside diminished transportation footprints, as vertical farms can be strategically situated closer to urban centres. Central to this innovation is the pivotal role of LED lighting, which provides the tailored light spectra essential for plant growth, while exhibiting superior energy efficiency and minimal heat emissions. Ongoing advancements in LED technology are instrumental in bolstering the sustainability and productivity of vertical farming systems [11,12].

Plant morphology, growth, and tissue differentiation are influenced by factors such as the quality, intensity, and duration of the photoperiod [13]. Plants exhibit non-uniform responses to all photosynthetically active radiation (PAR) (400–700 nm) wavelengths, with red (600–700 nm) and blue (420–460 nm) wavelengths proving most effective in driving photosynthesis [14,15].

Early research on the impact of artificial light on plants exploited broad-spectrum light sources and filters [16]. The advent of light emitting diodes (LEDs) has revolutionised horticulture by providing very narrow-beam monochromatic light for use as supplemental lighting or as the primary light source. Typically, a combination of red and blue LEDs is employed to offer the most efficient photosynthetically active radiation (PAR), and far-red (FR) (above 700 nm) light is often introduced to stimulate flowering. They play a critical role in photo-morphological development. Light wavelengths below 400 nm can have a potentially harmful effect on plant cell DNA. However, UV light can also enhance plant growth by promoting the production of protective compounds, such as flavonoids, and by improving stress tolerance. Moreover, UV exposure can boost the production of essential oils and pigments, leading to a higher quality and greater resilience to environmental stressors [11,17].

Prior research on the influence of LEDs and light wavelengths on *S. rebaudiana* has reported notable findings. The comparison between fluorescent lamps (FLs) and white LED lights (Philips LEDs) revealed no significant impact on the growth and yield of *S. rebaudiana* plants. However, the plants expressed a higher net photosynthesis value when grown under FL lamps compared to LEDs [10]. Conversely, the chemical composition of *S. rebaudiana* was notably affected by lighting conditions, with higher macro- and micronutrient contents observed when grown under LEDs, as opposed to fluorescent lamps [3,18,19,20,21].

Despite the existing research on light conditions and their effects on *S. rebaudiana* growth and yield, there is a need for a more comprehensive understanding of the influence of the wavelength on the plant’s physical properties, growth, and sweetener compounds. This study aimed to provide a greater understanding of the relationship of light spectra, including various combinations of blue, red, and UV wavelengths, with both the growth and quality of S. *rebaudiana.* The study also determined whether there was any effect on the specific sweetness components, such as stevioside and rebaudioside A, in the leaves of *S. rebaudiana.*

## 2. Methods and Materials

### 2.1. Source of Stevia rebaudiana, Plants and Cuttings

The experiment was conducted at the Cornwall plant factory at the University of Plymouth. The plant factory system at the University of Plymouth is designed to optimise the controlled environment for plant cultivation. It employs advanced hydroponic technology and LED lighting systems to maximise growth efficiency. The facility operates all year round, utilising precise control over the temperature, humidity, light spectrum, and nutrient supply, which allows for consistent hight-quality crop production, including *Stevia rebaudiana*. These controlled conditions not only enhance plant yield but also allow researchers to explore how environmental factors that influence the steviol glycoside content, contributing valuable insights into crop enhancement strategies. *Stevia rebaudiana* plants were obtained from Gardener’s Dream Ltd., Glasgow, UK (https://www.gardenersdream.co.uk/) (accessed on 20 April 2024). Cuttings were carefully taken from these plants, ensuring a 45-degree-angle cut to maximise the surface area. Cuttings were kept submerged in water until transplantation. Before the new cuttings were moved into 1-inch rock wool cubes, a rooting gel (Progrow Ltd., Plymouth, UK) was applied to each cutting to encourage the roots to grow faster. Rockwool cubes (Progrow, Ltd., UK) were fully immersed in water for 5 min, with this process repeated three times. Subsequently, the cubes were soaked in full-strength nutrient solution (VitaLink Max Grow A&B, Regrow, Exeter, UK) for another 5 min. A high-humidity environment was maintained by covering the tray with a lid. Cuttings remained in greenhouses (Skardon Garden, University of Plymouth) for two weeks before the lid was removed, ensuring that a good rooting level was established.

### 2.2. Transplantation and Light Treatments

**Figure 1:**
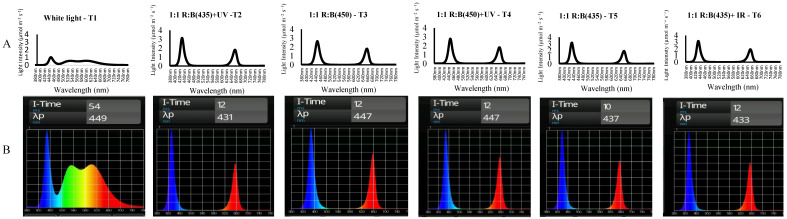
The spectra of the lighting treatments were assessed, using a UPRtek spectrophotometer, measuring (**A**) the radiant density of the light spectrum intensity and (**B**) the relative light intensity (T1: (50% cool white + 50% warm light), T2: Red: 663: Blue: 435 + UV365 1:1, T3: Red: B450 1:1, T4: Red: B450 + UV 1:1, T5: Red: B435 1:1, T6: Red: B435 + IR 1:1).

Once cuttings had rooted and were stable in the initial rockwool cubes, they were transplanted into 4-inch rockwool cubes (Figure 1). These plants were distributed across vertical racking under various light treatments. They were relocated to an Ebb and Flow hydroponic system within the plant factory at the University of Plymouth. This facility, originally a converted greenhouse, was adapted for plant cultivation and features a multi-tier hydroponic set-up system equipped with interchangeable LED light units, thereby eliminating external light. Divided into several multi-shelf hydroponic units, each with three tiers spaced 50 cm apart, the system provided optimal growth conditions. Temperature and humidity levels were meticulously monitored, using Gemini data loggers (Tinytag plus, part No GP-1590, Chichester, UK) and an instant thermometer (Fisher Scientific, Loughborough, UK), maintaining a temperature of 24 ± 2 °C and relative humidity of 65 ± 5%. The light–dark cycle was set at 16/8 h. Each irrigation bed received light from specific arrays of LEDs (Shenzhen Lumini Technology Co Ltd., Shenzhen, China), emitting different light spectra as follows: treatments T1: (50% cool white + 50% warm light), T2: Red: 663: Blue: 435 + UV365 1:1, T3 Red: B450 1:1, T4: Red: B450 + UV 1:1, T5: Red: B435 1:1, T6: Red: B435 + IR 1:1) (Figure 1). Light intensity was set at 160 µmol m^−2^ s^−1^ PPFD (photosynthetic photon flux density), and light spectra were measured using a handheld LED spectrophotometer (UPRtek MK350S) [11]. Plants were grown for five weeks before the harvesting stage, and four plants (replicates) were included in each light treatment.

### 2.3. Physiological and Growth Parameters

The chlorophyll fluorescence ratio (*Fv*/*Fm*) and performance index (PI) were measured using a Hansatech Pocket PEA meter from Hansatech Ltd. (Norfolk, UK) and scanning the second pair of leaves of each plant. The same pair of leaves was used to measure and determine the stomatal conductance (cm s^−1^) using a Delta-T AP4 Leaf Porometer (Delta T Devices, Cambridge, UK). Leaf temperature was measured and recorded using a Testo handheld infrared imaging camera (Testo 875-1i, Alton Hampshire, UK) on the second pair of leaves from the top for each plant, and the average temperature of the two leaves was used in statistical analysis before the lights were on. Measurements of leaf temperature were taken from plants at four weeks old [22,23,24].

**Figure 2:**
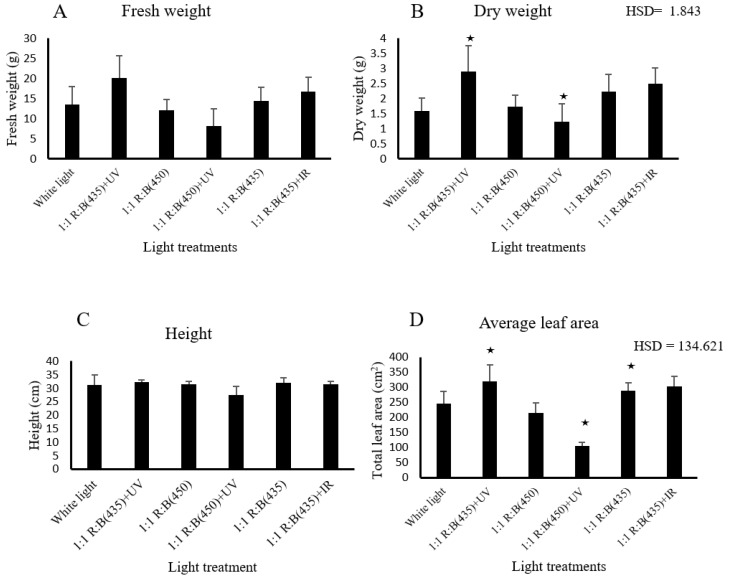
The effect of light treatment on growth: (**A**) fresh weight (g), (**B**) dry weight (g), (**C**) plant height (cm), (**D**) average leaf area of the top five leaves of *S. rebaudiana* at the harvest stage (5 weeks after obtaining the cuttings from mother plants). ★ means the treatment is ≥ HSD and there is a significant difference between treatments.

Plant growth responses were also measured in four plants from each treatment and three parameters, plant height (cm), fresh weight, and dry weight (g), were assessed. In addition, The average leaf area (cm2) of the top five leaves from each plant, per treatment, was measured using ImageJ software (version 1.53)., an open-source software (available to download from https://fiji.sc/) (accessed on 1 April 2024) [24,25,26]. The height of stevia was measured at two stages: firstly, after one week of placing the cuttings (plants) under the light treatments and, secondly, when the plants were harvested after five weeks (Figure 2).

#### 2.3.1. Chlorophyll Content Analysis

Chlorophyll content was measured using a method described by Porra [27]. Leaf samples of freeze-dried plant tissue weighing 10 mg were ground with 9 mL of 80% acetone. The total volume was adjusted to 10 mL with 80% acetone and then centrifuged for 3 min. Absorbance was measured against the 80% acetone blank. A glass cuvette containing 2 mL of the supernatant was used, and the absorbance was recorded at 664 nm (A_664_) and 647 nm (A_647_) using a Jenway 7315 spectrophotometer (Fisher Scientific, Loughborough, Leicestershire, UK). The formulas were determined based on the absorbance maxima of each pigment and were contingent on the solvent used. The samples were dissolved in acetone. The formulas were as follows:

Chlorophyll a: Ca, Chlorophyll b: Cb, Total chlorophyll: Total C.

Ca = 12.25 A_664_ − 2.55 A_647_

Cb = 20.31 A_647_ − 4.91 A_664_

Total C = 17.76 A_647_ − 7.34 A_664_

The readings were then converted to measure the chlorophyll content per gram of freeze-dried weight. Each sample (plant leaves) was freeze-dried, then ground and mixed. We took 0.2 g for each sample, with four replicates for each light treatment, following the methodology described by Porra [27].

#### 2.3.2. High-Performance Liquid Chromatography Analysis

The stevioside and rebaudioside A standards were sourced from Merck Life Science, Gillingham, UK. High-performance liquid chromatography (HPLC) solvents included liquid chromatography, grade acetonitrile, and methanol. Various solvents, including chloroform, water, acetonitrile, and water and acetonitrile mixed (8:2, *v*/*v* and 9:1, *v*/*v*) were employed to wash out impurities. Solvent wash volumes (5.0 mL) were eluted at the stationary phase. The optimal solvent effectively removed impure compounds without eluting the analytes. Each eluent’s optimisation of the washing solution was analysed by RP-HPLC. The stationary phase employed was Eurosphere C-18 (250 × 4.8 mm, 5 µm) with a guard column. All leaves from each plant were collected, ground, and thoroughly mixed to create a homogeneous sample. After adding water, the samples were further homogenized and mixed using ultrasonic technology. The mobile phase consisted of a mixture of methanol (10%) in water: acetonitrile (65:35, *v*/*v*). A flow rate of 0.6 mL/min was applied to the mobile phase. Separation was detected using a UV detector set at 200 nm. For each sample injection, 20 µL was introduced via a Pheodyne 7726i injector (Rheodyne Ltd, Turvey, UK). Four plant samples (each sample was from one plant) were taken and measured for each light treatment [28,29,30].

### 2.4. Statistical Analysis

For each light treatment, three replicates were performed, each consisting of four plants. The results are presented as the mean ± standard error (S.E.). Data were analysed using analysis of variance (ANOVA) in Minitab (version 19), and a mean comparison was made using the Tukey’s honestly significant difference (HSD) test at a 5% significance level (find the Supplementary Materials Tables S1–S10 for more details).

## 3. Results

### 3.1. Morphological Parameters

Plant fresh and dry weights exhibited minimal and statistically non-significant variation among the different light treatments, with corresponding *p*-values of 0.358 and 0.209, respectively (Figure 2). However, the analysis also revealed that light treatments significantly affect dry weight, with 1:1 R:B (435) + UV and 1:1 R:B (450) + UV treatments yielding higher weights compared to combinations including 1:1 R:B (435) + IR or white light (HSD = 1.843). These findings emphasise the influence of blue light (435 nm) and UV supplementation on dry biomass production of *S. rebaudiana.* Similarly, there was no significant difference between light treatments impacting the stevia heights at two stages. The first stage was the first week of transferring cuttings to Rockwool 4-inch tubes (the plants were 8 weeks old). The second stage was when the plants were harvested at five weeks after transplanting. The *p*-values were 0.782 and 0.571, respectively (Figure 2).

**Figure 3:**
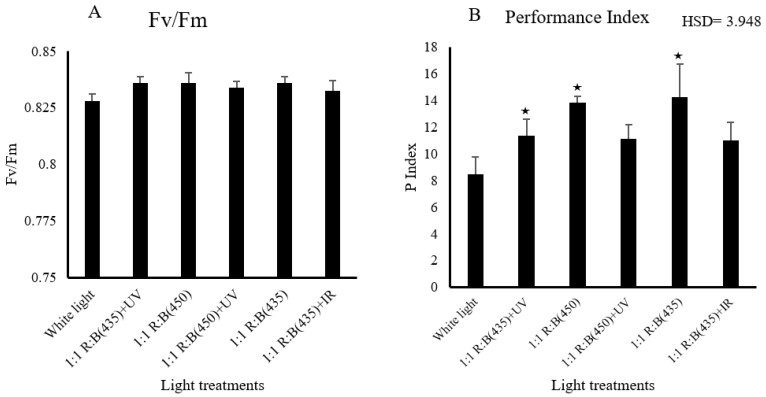
The effect of light treatment on health traits: (**A**) chlorophyll fluorescence parameter (*Fv/Fm*), (**B**) performance index of *S. rebaudiana* at the harvest stage. ★ means the treatment is ≥ HSD and there is a significant difference between treatments.

For plant health parameters, light treatments demonstrated a noteworthy influence on the performance index (*p* = 0.038), as illustrated in Figure 3. Specifically, the blue-rich spectrum with a wavelength of 435 nm, employed as a source of blue light, had a significant effect. In addition, the *p* index was significantly higher under the 1:1 R:B (450) treatment compared to white light (HSD = 3.948). Furthermore, 1:1 R:B (435) + UV showed significant differences comparing with 1:1 R:B (435) + IR (HSD = 3.948). In terms of the chlorophyll fluorescence parameter (*Fv/Fm*), there was no significant difference between the light treatments (*p* = 0.697). Both measurements are shown in Figure 3.

### 3.2. Physiological Parameters

**Figure 4:**
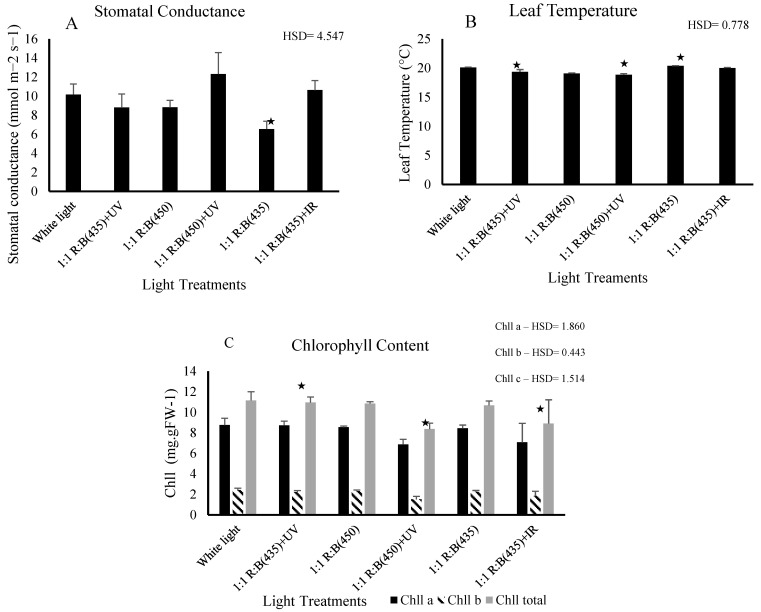
The effect of light treatments on the (**A**) chlorophyll content, (**B**) stomatal conductance, and (**C**) leaf temperature of *S. rebaudiana*. The impacts were significantly different in all 6 light treatments (*p* < 0.05) for the chlorophyll contents of Chll a, Chll b, and Chll c, stomatal conductance, and leaf temperature. ★ means the treatment is ≥ HSD and there is a significant difference between treatments.

The observed significant difference in chlorophyll content implies that certain light treatments stimulate or hinder chlorophyll production in stevia leaves. Significant differences in chlorophyll content were observed among the light treatments (*p* < 0.05), indicating that light spectra played a crucial role in influencing chlorophyll production in *S. rebaudiana*. Blue light (435 nm) and the combination of blue light (435 nm) with UV (365 nm) were particularly effective in enhancing chlorophyll A, chlorophyll B and total chlorophyll levels. Significant differences were found in chlorophyll A content (*p* = 0.022) and chlorophyll B content (*p* = 0.001) (Figure 4). The 1:1 R:B (450) + UV treatment showed significantly lower chlorophyll levels compared to white light (HSD = 0.443). For chlorophyll a, 1:1 R:B (450) + UV also differed significantly from the white light (HSD = 1.86). These findings suggest that specific LED light treatments can significantly improve photosynthetic efficiency, with potential implications for optimising growth conditions in controlled-environment agriculture (CEA) systems, difference in phenotype can be found in supplementary section (Figure S1).

The stomatal conductance of *S. rebaudiana* showed a significant variation between light treatments, demonstrating that different light spectra can affect physiological processes such as gas exchange. Stomatal conductance was measured, and the results revealed that the combination of blue light (435 nm) and UV light (365 nm) significantly increased the stomatal conductance compared to other treatments (*p* = 0.004). Notably, 1:1 R:B (435) + UV resulted in higher stomatal conductance compared to 1:1 R:B (450) + UV (HSD = 4.547). The highest stomatal conductance was observed under the blue (450 nm) and UV combination, followed by the treatment with red and blue light (435 nm). In contrast, the control group exposed to white light exhibited a significantly higher mean compared to 1:1 R:B (450) (HSD = 4.547). This suggests that a more targeted light spectrum, which includes blue UV wavelengths, can optimise gas-exchange progress by keeping stomata more open, which is crucial for photosynthesis and transpiration. Leaf temperatures in *S. rebaudiana* exhibited notable differences across the various light treatments, illustrating how specific light wavelengths can affect thermal regulation in plant tissues.

Leaf temperature was monitored using an FLIR thermal imaging camera, and significant differences in leaf temperature were observed among treatments (*p* < 0.05). The 1:1 R:B (435) + UV treatment exhibited a significantly lower leaf temperature compared to 1:1 R:B (435) and 1:1 R:B (435) + IR (HSD = 0.778). Furthermore, 1:1 R:B (450) + UV showed a significant difference compared to white light (HSD = 0.778). The coolest leaf temperatures were recorded in plants exposed to the blue and UV combination, followed by the treatment with red and blue light (450 nm). In contrast, plants grown under white light (control) displayed the highest leaf temperature, suggesting less efficient heat dissipation.

### 3.3. Sweetening Compound Analysis

This study explored the subtle impact of six different light treatments on the growth of stevia plants, using HPLC analysis to determine the concentrations of stevioside and rebaudioside A (reb A). The results demonstrated marked and statistically significant differences in the levels of these crucial sweetening components across all treatments, emphasising the substantial impact of light conditions on the phytochemical composition of stevia.

**Figure 5:**
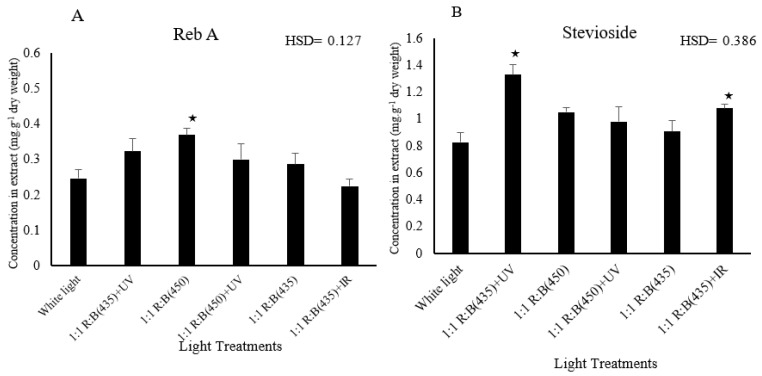
Effect of light treatment on the components of sweetness: reb A (**A**) and stevioside (**B**) of *S. rebaudiana.* ★ means the treatment is ≥ HSD and there is a significant difference between treatments.

**Figure 6:**
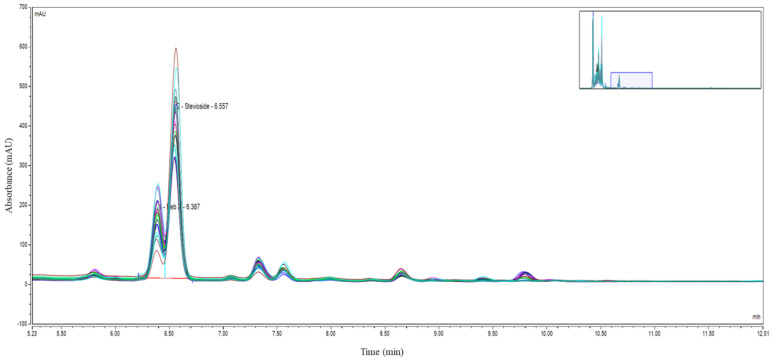
HPLC analysis showing the absorbance profiles of stevioside and reb A components over time.

A thorough analysis of stevioside and reb A concentrations, conducted with the use of HPLC, revealed significant differences across the various light treatments. Specifically, the stevioside area was significantly influenced by light treatments (*p* = 0.023). The 1:1 R:B (435) + UV treatment showed a significantly higher mean compared to white light (HSD = 0.386). Additionally, the treatment involving blue light at 435 nm, combined with UV supplementation, exhibited a distinct effect on the accumulation of stevioside and reb A, suggesting that certain wavelengths may stimulate or inhibit specific biosynthesis pathways related to these sweetening agents. As shown in Figure 5, Reb A concentrations varied significantly among treatments. Figure 6 presents the HPLC chromatograms, highlighting the retention times of stevioside and reb A.

## 4. Discussion

This study provides valuable insights into the effects of different LED light treatments on the growth, physiological parameters, and biochemical composition of *S. rebaudiana*. Increasing global demand for stevia as a natural sweetener and pharmaceutical resource necessitates a deeper understanding of the environmental factors influencing its productivity and quality. This investigation contributes to addressing that gap by exposing the intricate relationship between light spectra and stevia’s growth dynamics and biochemical properties [10,31,32,33]. Blue light plays a critical role in plant morphogenesis by activating specific photoreceptors, primarily cryptochromes and phytochromes [34]. These photoreceptors are instrumental in regulating processes such as stomatal opening, hypocotyl elongation, and orientation of leaves, which optimise light capture for photosynthesis. The importance of blue light in plant growth and physiological processes has been well-documented across various studies [35,36,37]. Rihan et al. [11] demonstrated that specific wavelengths can significantly influence plant morphology and physiological traits, supporting the findings of this study, in which blue light at 435 nm was found to increase the average leaf area and potentially enhance the synthesis of secondary metabolites in *Stevia rebaudiana*. In stevia, exposure to blue light has previously been shown to significantly enhance leaf expression and stem elongation. This effect is mediated by the stimulation of cryptochromes, which not only regulate growth but also entrain circadian rhythms that control physiological processes at the cellular level [1,38,39].

The results reveal notable variations in stevia’s growth parameters and physiological responses under different LED light treatments. Plant biomass (fresh weight) exhibited minimal variance across treatments, and the data indicate that 1:1 R:B (435) + UV and 1:1 R:B (450) + UV treatments yielded higher dry weights than 1:1 R:B (435) + IR or white light, confirmed by Tukey’s HSD (HSD = 1.843). Similarly, plant height measurements at both stages did not significantly differ among treatments. However, the average leaf area of the top five leaves was strongly influenced by the spectral quality, with the combination of blue light (435 nm) and UV (365 nm) significantly enhancing leaf expansion, while 1:1 R:B (450) + UV yielded a notably lower average leaf area (HSD = 134.62). These discoveries align with previous research suggesting that white light positively affects the growth and development of plants, surpassing even the impact of blue light when added to red light LEDs [10,39,40,41,42].

Moreover, the significant differences in leaf temperature observed among light treatments highlight the intricate interplay between light conditions and thermal regulation in stevia plants. These variations can be attributed to the differential absorption and utilisation of the light energy based on the chlorophyll content and stomatal conductance, leading to fluctuations in metabolic activity and heat production within the leaf tissues, which enhance the capacity for the neutralisation of reactive oxygen species through heightened activity of reactive enzymes or increased concentrations of non-enzymatic antioxidants [10,27,43,44].

The performance index, a crucial indicator of plant health and vitality, showed significant variability across light treatments, with blue-rich spectrum light (435 nm) exerting a notable influence. This suggests that certain light spectra may optimise photosynthetic efficiency and overall plant performance. Conversely, chlorophyll fluorescence parameters (*Fv*/*Fm*) remained unaffected by light treatments, indicating that variations in light spectra may not significantly alter chlorophyll activity or efficiency, which matches the findings of recent research [33,45,46,47,48,49,50].

The exposure of *Stevia rebaudiana* to specific spectra of LED light, notably blue and UV, has profound implications for its biochemical composition, especially the enhancement of its primary sweetening compounds, stevioside and rebaudioside A. These compounds are synthesised through a complex pathway that converts the precursor molecule, steviol, into various steviol glycosides [51,52]. Moreover, research indicates that specific light wavelengths can induce the expression of key enzymes involved in this pathway, such as kaurenoic acid hydroxylase, which play a crucial role in the early steps of steviol glycoside biosynthesis. The results reveal marked and statistically significant differences in the levels of these crucial sweetening components across all treatments, highlighting the substantial impact of light conditions on the phytochemical composition of stevia, as mentioned by many researchers with regard to these sweetness components [53,54].

HPLC allowed for a precise quantification of these compounds by measuring peak heights and areas on the chromatograms, reflecting the relative concentrations of stevioside and reb A across the range of treatments. These variations indicate that light wavelengths can be carefully selected to enhance the yield and quality of stevia-derived sweeteners, offering valuable insights into optimising cultivation practices for the desired phytochemical profile [51,55]. To expose these variations further, employing HPLC techniques, a comprehensive analysis of stevioside and reb A concentrations was conducted, uncovering noteworthy variations among the different light treatments. Particularly notable were the results obtained from the treatment involving blue light at 435 nm with UV supplementation, which exhibited a significant impact on the profiles of these key sweetening compounds. Quantification of peak heights and areas enabled the assessment of the relative concentrations of stevioside and reb A across the various light treatments; these findings are similar to the outcomes from [56,57].

The results demonstrated the complexity of how stevia plants respond to light, especially in terms of phytochemical production. Stevioside and reb A biosynthesis appear sensitive to the spectral quality of light, suggesting that optimizing light treatments can be assessed using Tukey’s Honestly Significant Difference test. These findings are consistent with previous outcomes from [56,58].

The results underscore the complexity of the manner in which stevia plants respond to different light treatments. The Tukey’s Honestly Significant Difference test for multiple comparisons could become an essential part of stevia cultivation practises, thereby offering industrial producers the capacity to enhance sweetness levels and product quality, leading to more efficient and profitable production [59,60]. These findings highlight the need for additional research to pinpoint the exact mechanisms through which different light wavelengths impact biosynthesis in stevia plants. Understanding these mechanisms at a biochemical level will allow researchers to refine light treatments further, offering greater control over the production of stevioside and reb A. Our study supports the view that the use of specialised light spectra has the potential to enhance plant quality. It suggests that, with the insights gained from further research, producers could apply tailored light regimes to optimise stevia cultivation and fully exploit its potential in the natural sweetener market [32,47,55,56,61,62,63].

## 5. Conclusions

This study underscores the significance of LED lighting in shaping the growth and biochemical composition of *S. rebaudiana*. By elucidating the nuanced effects of different light spectra on stevia’s physiological and biochemical responses, this research contributes to the development of sustainable cultivation practices and the production of high-quality stevia-derived products. Understanding the mechanisms underlying light-mediated responses in stevia is necessary and important to further leverage the potential of LED lighting in enhancing its productivity and nutritional properties [32,44,50,55,63,64,65,66,67,68].

## Data Availability

The original contributions presented in this study are included in the article. Further inquiries can be directed to the corresponding authors.

## Supplementary Materials

Supplementary materials provided with this study include a spreadsheet containing the data analysis (Tables S1–S10) and the Figure S1 detailing the phenotype results. The following supporting information can be downloaded at: https://www.mdpi.com/article/10.3390/biology14020108/s1, Figure S1. Phenotype; Table S1. Leaf temperature; Table S2. Height data; Table S3. P index data; Table S4. Fv/Fm data; Table S5. Leaf area data; Table S6. Fresh weight data; Table S7. Dry weight data; Table S8. Stomatal conductance data; Table S9. stevioside data (height and area); Table S10. Reb A data.
