# Supplementary figures and images for: The Impact of LED Light Spectra on the Growth, Yield, Physiology, and Sweetness Compound of Stevia rebaudiana
Source: Biology (Basel). 2025 Jan 21;14(2):108. doi: 10.3390/biology14020108 (PMC11852103; doi:10.3390/biology14020108)

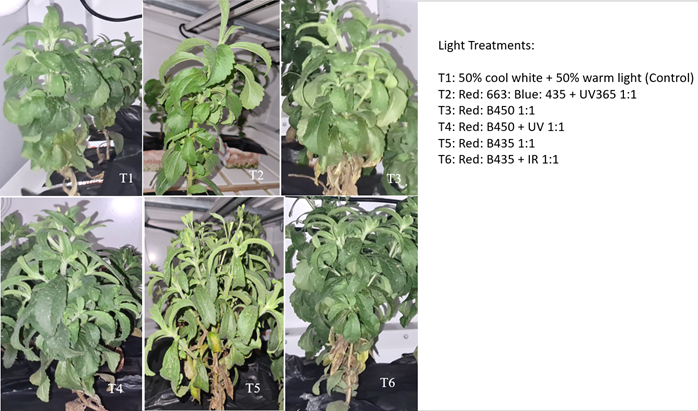

Supplement: Supplementary file 1 [file biology-14-00108-s001.zip › Figure S1. Phenotype.png]
